# Development of indirect enzyme-linked immunosorbent assay for detection of porcine epidemic diarrhea virus specific antibodies (IgG) in serum of naturally infected pigs

**DOI:** 10.1186/s12917-019-2123-2

**Published:** 2019-11-12

**Authors:** Ohnmar Myint, Ayako Yoshida, Satoshi Sekiguchi, Nguyen Van Diep, Naoyuki Fuke, Uda Zahli Izzati, Takuya Hirai, Ryoji Yamaguchi

**Affiliations:** 1 Department of Veterinary Pathology, Faculty of Agriculture, University of Miyazaki, 1-1 Gakuenkibanadai-Nishi, Miyazaki, 889-2192 Japan; 2 Department of Veterinary Parasitic Diseases, Faculty of Agriculture, University of Miyazaki, Miyazaki, Japan; 3 Center for Animal Disease Control, University of Miyazaki, Miyazaki, Japan; 4 Department of Animal Infectious Disease and Prevention, Faculty of Agriculture, University of Miyazaki, Miyazaki, Japan

**Keywords:** Porcine epidemic diarrhea virus, (PEDV), ELISA, Neutralization test, Pre-coated plate, Long-term preservation

## Abstract

**Background:**

Porcine epidemic diarrhea virus (PEDV) infection is a highly contagious infectious disease causing watery diarrhea, vomiting, dehydration and high mortality rate in newborn piglets. PEDV infection can cause high economic losses in pig industry. In Japan, a PEDV outbreak occurred with high mortality from 2013 to 2015. Even though until now, PEDV infection occurs sporadically. For the control and monitoring of PEDV infection, not only symptomatic pigs, but also asymptomatic pigs should be identified. The objective of this study is to develop and optimize novel indirect ELISA as a simple, rapid, sensitive and specific method for the detection of anti-PEDV antibodies and evaluate the efficacy of the assay as a diagnostic method for PED.

**Results:**

One hundred sixty-two serum samples, consisting of 81 neutralization test (NT) positive and 81 NT negative sera, were applied to the assay. Indirect ELISA test based on whole virus antigen (NK94P6 strain) derived from Vero cell culture was evaluated by receiver operating characteristic (ROC) analysis with neutralization test (NT) as a reference method, and cut-off value was determined as 0.320 with sensitivity and specificity of 92.6 and 90.1%, respectively. The area under curve (AUC) was 0.949, indicating excellent accuracy of indirect ELISA test. There was significant positive correlation between indirect ELISA and neutralization test (*R* = 0.815, *P* < 0.05). Furthermore, the kappa statics showed the excellent agreement between these two tests (kappa value = 0.815). In addition, the sensitivity and specificity of preserved plates with different periods (1 day, 2 weeks, 1, 2, 3, 4, 5 and 6 months) after drying antigen coated plates were 100% and 80–100%, respectively.

**Conclusions:**

The developed indirect ELISA test in our study would be useful as a reliable test for serological survey and disease control of PEDV infection, and our pre-antigen coated ELISA plates can be preserved at 4 °C until at least 6 months.

## Background

Porcine epidemic diarrhea is caused by porcine epidemic diarrhea virus (PEDV) that belongs to genus *Alphacoronavirus*, family *Coronaviridae*, order *Nidovirales* [1, 2]. PEDV infection is a highly contagious infectious disease and is characterized by watery diarrhea, and vomiting leading to dehydration [3]. PEDV can infect all ages of pigs and the exhibition of clinical signs vary according to the age of pigs [4]. Morbidity and mortality rate of PEDV infection may reach up to 100% in piglets but variable in adult pigs [5]. Nowadays, PEDV infection has become endemic in North and South America, Asia and Europe, causing significant economic losses in the worldwide swine industry [3, 6].

The incubation period of PEDV is 1–4 days [7]. Since anti-PEDV IgG antibodies in the serum can be detected 13 days after inoculation of virus [8], serum samples for the detection of PEDV specific antibodies should be collected 2–3 weeks after the onset of diarrhea. These PEDV IgG antibodies in ELISA test persist in the serum for at least 1 year after post infection [2] and the virus neutralization titers for PEDV in plasma remains high beyond 6 months post-infection [9].

There are two kinds of diagnostic methods; virological and serological, for PEDV infection in pigs. In virological methods, virus isolation, immunofluorescence assay, immunohistochemistry test, polymerase chain reaction based assays and isothermal amplification assays are used for detection of virus, its nucleic and viral protein. In serological methods, indirect immunofluorescence assay, viral neutralization assays, fluorescent microsphere immunoassay, blocking Enzyme-linked Immunosorbent Assay **(**ELISA) and indirect ELISA by using virus structural protein are used for detection of anti-PEDV specific antibodies [10]. Compared with virological methods, serological tests are cost-effective and can detect viral specific antibodies prior exposure in the absence of virus infection [11].

PED sporadic outbreak still occurs in Japan. For the control of PED, not only symptomatic pigs, but also asymptomatic pigs should be detected to confirm whether PEDV infection is still present or not. Serum Neutralization test (NT) is the gold standard of serological assay for PEDV specific antibodies detection due to its high specificity in Japan. However, this test is laborious and, time-consuming, requiring manual result reading, interpretation of virus induced cytopathic effect endpoints and reduction of NT titer due to virus mutation [12, 13], while ELISA test is simple, easy, rapid and accurate and can screen large number of serum samples [9, 11]. To apply for the monitoring and the surveillance of PEDV infection, indirect ELISA is more preferable for PED detection. The objective of this study is the development and optimization of indirect ELISA assay for the detection of anti-PEDV antibodies and evaluate the efficacy of indirect ELISA test as a diagnostic method for PEDV infection.

## Results

### Validation of novel indirect ELISA

**Fig. 1:**
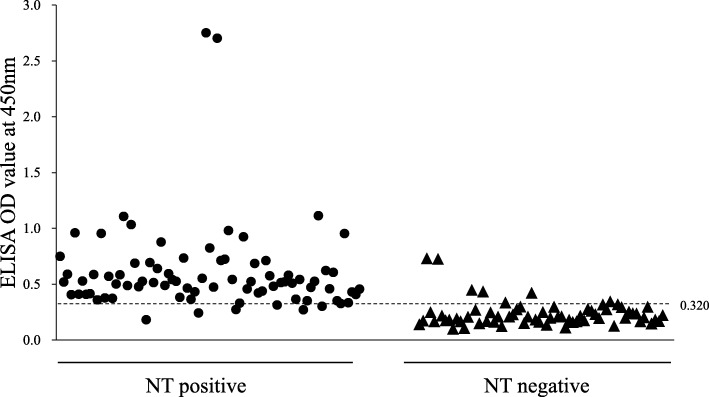
Reactivity of pig sera with PED whole virus antigen in indirect ELISA. NT: serum neutralization test; ELISA: Enzyme linked-immunosorbent assay. Dotted line represents cut off value for ELISA test. 81 NT positive and 81 NT negative sera were assesed in our developed indirect ELISA. The sera above dotted line considered PEDV positive and sera below dotted line were PEDV negative according to cut off value for ELISA test

Eighty-one PEDV NT positive samples and 81 PEDV NT negative samples were tested by indirect ELISA based on whole viral antigen (Fig. 1). The results of indirect ELISA were evaluated by ROC analysis with neutralization test as a reference method. In the ROC analysis, the area under curve (AUC) was determined at 0.949. When the cut-off value was set at 0.320 based on the ROC analysis, the sensitivity and specificity of this ELISA were 92.6 and 90.1%, respectively. As shown in Table 1, indirect ELISA produced 6 false negatives (7.4%) and 9 false positives (11.1%).

**Table 1:** Comparison of PEDV seropositive result with indirect ELISA and neutralization test

		ELISA		Total
		Positive	Negative	
NT	Positive	75 (92.6%)	6 (7.4%)	81
	Negative	9 (11.1%)	72 (88.9%)	81
	Total	84	78	162

*NT* Neutralization test, *ELISA* Indirect enzyme linked immunosorbent assay

### Correlation between indirect ELISA and neutralization test

Significant positive correlation between indirect ELISA and neutralization test in the PEDV specific antibodies detection were observed (*R* = 0.815, *P* < 0.05). There was almost perfect agreement between indirect ELISA and neutralization test (kappa value = 0.815). The results showed that maximum agreement was obtained from 1: ≥ 16 (100%) of neutralization titer and minimum agreement was obtained from 1: 2 (85.2%) of neutralization titer (Table 2).

**Table 2:** Consistency between indirect ELISA and NT in anti- PEDV antibody detection

Neutralization test	Total tested samples	Indirect ELISA		% Agreement
		Positive	Negative	
≥ 64	2	2	0	100
32	2	2	0	100
16	11	11	0	100
8	19	18	1	94.7
4	20	19	1	95
2	27	23	4	85.2
≤ 2	81	9	72	88.9

### Detection for cross-reactivity of antibodies against other common porcine viruses

**Fig. 2:**
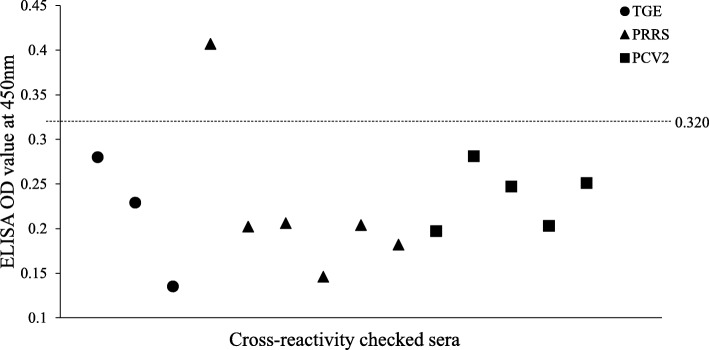
Cross-reactivity of other common swine viruses’ antibodies with ELISA PEDV antigen. Dotted line represents cut off value for the ELISA. 3 TGE, 6 PRRS and 5 PCV2 antibodies positive sera were used for cross-reactivity detection in the PEDV indirect ELISA. All of the sera showed no cross-reactivity except only one PRRS seropositive sample showed cross-reactivity with ELISA PEDV antigen

The antibodies positive sera for TGEV, PRRSV and PCV2 were tested by using the PEDV indirect ELISA for evaluation of cross-reactivity. There was no cross-reactivity with antibodies of these common porcine viruses except for one PRRSV seropositive sample in this indirect ELISA according to the result (Fig. 2).

### Long term preservation of antigen coated plates for the detection of anti-PEDV antibodies

**Fig. 3:**
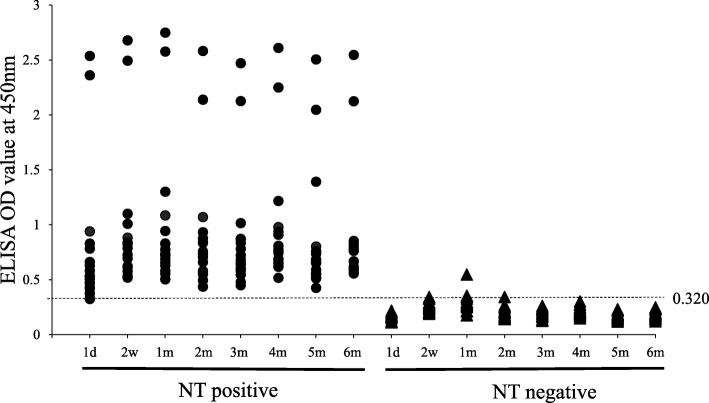
Reactivity of sera to PEDV antigen in indirect ELISA using preserved antigen coated plates. d: day; w: week; m: month. Dotted line shows positive/ negative cut off value of ELISA. Number of PEDV positive and negative sera were not different between each plates even though time were difference after antigen coating and drying ELISA plates

The ELISA plates were coated with diluted whole viral antigen and blocked with blocking solution. After that, these plates were dried at 37 °C for 4 h and kept at 4 °C until used. The stability of antigen coated plates was investigated at 1 day, 2 weeks, 1, 2, 3, 4, 5 and 6 months after drying the plate. The same 17 PEDV NT positive and 20 PEDV NT negative sera were used for assay in each time point. The OD values of each serum sample from the assays in the different time-points are shown in Fig. 3. Statistically, there were no significant differences (*P* > 0.05) in the number of PED positive/negative sera and OD value between each plate from 1 day to 6 months after drying the plate. The sensitivity and specificity of each ELISA plate stored at 4 °C for different periods after drying plates are also described in Table 3. The sensitivity of all preserved plates were 100% and specificity of them ranged from 80 to 100%.

**Table 3:** Sensitivity and specificity of preserved ELISA plates after antigen coating and drying plates

	1 d	2 w	1 m	2 m	3 m	4 m	5 m	6 m
Sensitivity (%)	100	100	100	100	100	100	100	100
Specificity (%)	100	85	80	95	100	100	100	100

*d* day, *w* week, *m* month

## Discussion

In Japan, the first PED like disease outbreak occurred in late 1982 and early 1983 [14, 15] and became pandemic in 1993–1996 [14, 16]. After that, the PEDV outbreak occurred again in 1996 and PEDV infection become sporadic. From late 2013 to 2015, a PEDV infection re-emerged with high mortality. Currently, PEDV infections occur sporadically in Japan (Ministry of Agriculture, Forestry and Fisheries of Japan). It has been reported that pig farms with no PEDV clinical signs contained subclinical PEDV seropositive animals, which were detected by NT [17]. Considering this situation for controlling PED, we need to survey and monitor the PEDV infection. NT is useful in sero-surveillance of both recent and post PEDV infection [17]. However, NT has some disadvantages such as being laborious, time consuming and mutated neutralizing related epitope. The development of a simple, rapid, specific and economic technique is urgently needed for the detection of PEDV infection. Therefore, indirect ELISA for anti-PEDV antibody detection was developed and optimized the accuracy of assay in this study.

Serological assays, especially ELISA, were used widely for PEDV specific antibodies detection. The indirect ELISA differs based on coated antigen such as Vero cell culture derived from whole viral antigen and recombinant polypeptides derived from PEDV structural proteins (nucleocapsid, spike, membrane, and envelope) [8, 11, 13, 18–21].

Recombinant PEDV structural protein based indirect ELISA have been developed. Validation of indirect ELISA tests experienced variation and experimentally infected serum was used to evaluate the validity of test. Recombinant PEDV structural protein based ELISAs increase specificity but sensitivity can reduce due to heterogenicity of PEDV isolates [22, 23]. On the other hand, whole virus coated ELISA can produce high background, cross- reactivity with other viral antibodies and low specificity [11, 18]. However, the purified whole virus antigen involves all virally expressed proteins that increases sensitivity [24]. Moreover, whole viral antigen coated indirect ELISA is more sensitive than the PEDV structural protein based ELISA in the detection of heterogeneous strain [25]. Based on these advantages, whole viral antigen was used as an ELISA antigen in this study in order to improve the detection sensitivity for field serum samples. Whole NK94P6 PED virus was used as a coated antigen in the present study because this strain is gold standard strain in Japan.

Since NT is considered the gold standard among serological tests, we used NT as a reference method for comparison with indirect ELISA. Based on the ROC analysis, cut-off value was determined at 0.320. Sensitivity and specificity of this assay are 92.6 and 90.1%, respectively. Previously, it was reported that the sensitivity and specificity of indirect ELISA using whole viral antigen (KPEDV-9) were 89.1 and 94.5% [18]. Other researcher described that sensitivity and specificity of group 1a whole viral antigen (CV777) coated competitive ELISA were 93.5 and 91.2% [23]. The sensitivity and specificity of these reports were very similar with our result, even though the virus strain, antigen preparation methods and antigen detection techniques were different.

In addition, results indicated a high correlation (*R* = 0.815, *P* < 0.05) and almost perfect agreement (kappa value = 0.815) between indirect ELISA and neutralization test. Considering these results, this indirect ELISA would be useful for PEDV specific antibodies detection, equal to NT.

In this study, the assay produced 6 false negative (7.4%) and 9 false positive serum samples (11.1%). Regarding the 6 false negative samples, they may have occurred due to neutralization test detect both IgM and IgG although the ELISA test can detect only IgG [18]. This ELISA also showed 9 false positive serum samples. One possibility is that cross reactivity might have occurred with other protein in this ELISA, resulting the omission of virus purification step in this study. Another possibility is that indirect ELISA is more sensitive than the NT. There may be reduction of NT titer due to virus mutation in neutralizing epitope. It is likely that, NT and ELISA test results would not be identical because ELISA can detect PEDV specific antibodies other than the virus neutralization antibodies.

The results from this study showed that the maximum agreement was obtained from ≥1: 16 (100%) and minimum agreement was obtained from 1: 2 (85.19%) of neutralization titer. In this study, sera with ≥1:2 NT titer were considered PEDV NT positive. However, other studies described that the NT titer for seropositive were ≥ 1:4 and ≥ 1:8, respectively [18, 25]. If serum samples with ≥1:4 NT titer were applied as a NT positive sample, sensitivity and specificity of our indirect ELISA would increase.

Moreover, no cross-reactivity with antibodies against TGEV, PRRSV and PCV2 indicated that the developed PEDV indirect ELISA is effective and specific serological method for PEDV specific antibodies detection. However, only one PRRSV seropositive sample cross-reacted with ELISA antigen (PEDV) but the OD value was near to the cut off value. This would be due to non-specific binding of antibodies in this ELISA. Non-specific binding of antibodies in indirect ELISA is a common problem because specific or non-specific antibodies bound in the well [23, 26].

In addition, the stability of PEDV antigen coated plates was also investigated. According to the assay results using preserved pre-antigen coated ELISA plate, not only the number of PEDV positive and negative sera but also OD values of each serum between each plate were not significantly different. The sensitivity and specificity of the plate was 100% at 6 months after antigen coating and drying plate (Table 3). Therefore, our pre-antigen coated ELISA plate can be used and stored at 4 °C until at least 6 months. Our ELISA could be applicable in large-scaled of serological survey in the absence of active infection. Further studies will need to be conducted to investigate the seroprevalence of PEDV infection by applying this indirect ELISA.

## Conclusions

Developed indirect ELISA using Vero cell derived whole viral antigen is simple, useful, specific and reliable for serological survey and disease control management of PEDV infection. Moreover, our pre-antigen coated ELISA plates can be applied until 6 months when stored at 4 °C, the same as commercial ELISA kits.

## Methods

### Virus

PEDV strain NK94P6 was kindly provided by the National Institute of Animal Health, Japan. PEDV NK94P6 strain was used as a coated antigen for indirect ELISA plates in this study. This strain involved in classical clade group 1 of PEDV stain classification [17, 27].

### Serum samples collection

Blood samples were collected from growing-finishing pigs at two slaughterhouses, located on Kyushu Island from June to July 2014. Samples were collected from pigs aged over 6 months to avoid maternal antibodies. Information of farm status such as history of clinical signs of PEDV infection and reverse transcription polymerase chain reaction (RT-PCR) detection result were obtained from the meat inspection office of each slaughterhouse. Based on the information, 333 samples were collected from 16 PED positive farms and 1223 samples were collected from 64 farms showing no PED clinical sign. All of collected blood samples were centrifuged at 2500×g for 5 min and stored at − 20 °C. All of samples were subsequently tested by NT. In this study, 81 NT positive and 81 NT negative sera were used for the optimization and evaluation of indirect ELISA. Three transmissible gastroenteritis virus (TGEV) antibody positive sera provided from Miyazaki prefecture, 6 porcine reproductive and respiratory syndrome virus (PRRSV) antibody positive sera from our laboratory and 5 porcine circovirus type 2 (PCV2) seropositive sera kindly supplied by Professor Takami Okabayashi (University of Miyazaki, Japan) that were used for evaluation of cross-reactivity with the ELISA PEDV antigen. The antibody positive sera for TGEV and PRRSV were collected from naturally infected pigs and for PCV2 were collected from PCV2 vaccinated pig farm. Anti-TGEV antibodies were checked by neutralization test and anti-PRRSV and anti-PCV2 antibodies were tested by ELISA.

### Neutralization test

The neutralization test procedure was described previously. The cut off value for NT PEDV positive titer was described at ≥1:2, which follows the Japanese National Institute of Animal Health’s guide-lines [17].

### Preparation of PED whole viral antigen for ELISA

Vero cells (KY-5) was kindly provided by the National Institute of Animal Health, Japan. Vero-KY5 cells were cultivated in 75 cm^2^ flask with D-MEM (Dulbecco’s Modified Eagle’s Medium, Wako, Osaka, Japan) supplemented with 10% fetal bovine serum (FBS) and 100 U/ml penicillin-streptomycin (Wako, Japan) at 37 °C in a humidified atmosphere containing 5% CO_2_ for 2 to 3 days [17, 27].

When Vero-KY5 cells obtained 90% confluence, 6 ml of PEDV NK94P6 (2.5 × 10^5^TCID_50_/ml) was added to a flask and incubated for 1 h (hr) at 37 °C in a humidified atmosphere containing 5% CO_2_. 100 ml of maintenance solution (0.3% tryptose phosphate broth (TPB, Becton, Dickinson and company, USA), 0.02% yeast, 5 μg/ml of trypsin and D-MEM) were then added to the flask and placed into 5% CO_2_ incubator at 37 °C for 3 days and 10 h. When 100% CPE showed, the fluid was frozen, thawed and sonicated for 15 min. After centrifugation at 4000 rpm for 10 min at 4 °C, supernatant was taken and kept at − 70 °C. The virus titer was 4 × 10^5^ TCID_50_ /ml.

Amicon ultra-15 centrifugal filter 100 K devices (Merck Millipore Ltd., County Cork, Ireland), were applied for concentration of the virus solution. Approximately 12 ml of viral solution was added into 15 ml Amicon tube and centrifuged at swinging-bucket rotor at 4000×g for 20 min at 25 °C. The concentrated viral solution was collected and kept at − 70 °C until used. This solution was used as an ELISA antigen. The virus titer was 1 × 10^6^ TCID_50_/ml and 2.8 × 10^7^ copies of PEDV per 1 μl volume, determined by real time RT- PCR [28]. The protein concentration was 4.48 mg/ml, which was measured by Nano drop (Thermo Scientific, Thermo Fisher Scientific Inc., Waltham, MA).

### ELISA procedure

ELISA conditions were optimized using the serial dilutions of antigen and sera (Additional file 1: Figure S1 and Additional file 2: Figure S2). The whole viral antigen was diluted at 1:100 with coated buffer solution (0.05 M carbonate buffer, pH 9.6). Ninety-six wells ELISA plates (Nunc, Thermo Fisher Scientific Inc.) were coated with 50 μl of this solution and incubated at 4 °C, overnight. The plates were washed with 300 μl of washing buffer (0.05% Tween PBS) 4 times, blocked with 150 μl of blocking reagent for ELISA (Cosmo Bio, Tokyo, Japan) and incubated at room temperature (RT) for 2 h. After incubation, blocking reagent was completely aspirated and the plates were dried at 37 °C for 4 h. Then, all of the plates were sealed and kept at 4 °C until used.

All ELISA plates were allowed at RT before using them. The serum samples were diluted at 1:1000 with 1% casein Tris-buffered saline (TBS) and added into each well (50 μl/well). After incubation at 37 °C for 1 h, the wells were washed 4 times with washing buffer (300 μl/well), added with 1:5000 diluted goat anti-pig IgG, horseradish peroxidase conjugate (KPL, Gaithersburg, USA) and incubated at 37 °C for 1 h. The contents of wells were then aspirated and washed 4 times with washing buffer (300 μl/well). For color development, 50 μl of Tetramethylbenzidine substrate (TMB, KPL, Gaithersburg, USA) was added into each well and incubated at RT for 20 min in the dark. Finally, the reaction was stopped with 2 N-H_2_SO_4_ solution (Wako, Osaka, Japan) and read the optical densities (OD) by using 450 nm filter with 620 nm as a reference filter in Microplate Reader (Bio-Rad Laboratories, Hercules, CA, USA).

### Data analysis

Sensitivity, specificity and cut-off value of this ELISA test were analyzed by receiver operating characteristic (ROC) analysis. The results of the ELISA test and NT were analyzed by correlation analysis and described as Spearman’s R and Kappa value. ELISA stability test based on the number of positive/negative sera and OD value between each plate after drying were analyzed by Chi-square test and ANOVA test. All analyses were performed by computer programming language R (version 3.4.3; R development core team, Vienna, Australia) [29]. The interpretation of Kappa value was determined by ranges from 1 (complete agreement) to 0 (agreement is equal to that expected by chance). Kappa values are > 0.81: almost perfect agreement; 0.61–0.80: substantial agreement; 0.41–0.60: moderate agreement; 0.21–0.40: fair agreement; 0–0.20: slight agreement and 0: poor agreement [30].

## Supplementary information

**Additional file 1: Figure S1.** Evaluation for working antigen concentration of PEDV. For optimization of working PEDV antigen concentration, 3 NT positive sera (1: 128, 1:64, 1:8 of NT titer) and 3 NT negative sera (1:< 2 of NT titer) were tested at 1:100, 1:200, 1:400 and 1:800 dilutions of PEDV antigen coated ELISA plate. The serum samples were diluted at 1:1000 in this experiment. The highest OD value in NT positive sera and the lowest OD value in NT negative sera were observed for 1:100 dilution of PEDV antigen. Therefore, the working PEDV antigen concentration for the indirect ELISA was set as 1:100 dilution in this study.

**Additional file 2: Figure S2.** Evaluation for working serum samples dilution. Three NT positive (1: 128, 1:64, 1:8 of NT titer) and 3 NT negative sera (1:< 2 of NT titer) were diluted to 1:62.5, 1: 125, 1: 250, 1:500, 1:1000 and, 1:2000 and tested using 1:100 diluted PEDV antigen coated plates. All NT negative sera diluted more than 1:1000 gave OD value less than 0.3, and NT positive sample with NT titer (1: 8) showed OD value higher than 0.5 in 1:1000 dilution or less. Therefore, the working serum dilution for the indirect ELISA was set as 1:1000 in this study.

## Data Availability

The data supporting the conclusions of this article are included in this article. All data sets can be requested from correspondence with the authors.

## Abbreviations

AUC: Area under curve
CPE: Cytopathic effect
D-MEM: Dulbecco’s modified eagle’s medium
ELISA: Enzyme- linked immunosorbent assay
FBS: Fetal bovine serum
hr: Hour
Ig G: Immunoglobulin G
NT: Serum neutralization test
OD value: Optical density value
PBS: Phosphate buffer saline
PCV2: Porcine Circovirus type 2
PEDV: Porcine epidemic diarrhea virus
PRRSV: Porcine reproductive and respiratory syndrome virus
ROC: Receiver operating characteristic
RT: Room temperature
RT-PCR: Reverse transcription polymerase chain reaction
TBS: Tris-buffered saline
TCID: Tissue culture infective dose
TGEV: Porcine transmissible gastroenteritis virus
TPB: Tryptose phosphate broth

## References

[1] Pensaert M, De Bouck P (1978). A new coronavirus-like particle associated with diarrhea in swine. Arch Virol.

[2] Song D, Park B (2012). Porcine epidemic diarrhoea virus: a comprehensive review of molecular epidemiology, diagnosis, and vaccines. Virus Genes.

[3] Stevenson GW, Hoang H, Schwartz KJ, Burrough ER, Sun D, Madson D, Cooper VL, Pillatzki A, Gauger P, Schmitt BJ (2013). Emergence of porcine epidemic diarrhea virus in the United States: clinical signs, lesions and viral genomic sequences. J Vet Diagn Investig.

[4] Puranaveja S, Poolperm P, Lertwatcharasarakul P, Kesdaengsakonwut S, Boonsoongnern A, Urairong K, Kitikoon P, Choojai P, Kedkovid P, Teankum K, Thanawongnuwech R (2009). Chinese-like strain of porcine epidemic diarrhea virus, Thailand. Emerg Infect Dis.

[5] Saif LJ, Pensaert MB, Sestak K, Yeo SG, Jung K, Zimmerman JJ, Karriker LA, Ramirez A, Schwartz KJ, Stevenson GW (2012). Porcine epidemic diarrhea virus. Diseases of swine.

[6] Chaochao LV, Xiao Y, Li X, Tian K (2016). Porcine epidemic diarrhea virus: current insights. Virus Adapt Treat.

[7] World Organization for Animal Health (2014). OIE Technical Factsheet, Infection with porcine epidemic diarrhea virus.

[8] Bjustrom-Kraft J, Woodard K, Gimenez-Lirola L, Rotolo M, Wang C, Sun Y, Lasley, Zhang J, Baum D, Gauger P, Main R, Zimmerman J (2016). Porcine epidemic diarrhea virus (PEDV) detection and antibody response in commercial growing pigs. BMC Vet Res.

[9] Ouyang K, Shyu DL, Dhakal S, Hiremath J, Binjawadagi B, Lakshmanappa YS, Guo R, Ransburgh R, Bondra KM, Gauger P, Zhang J, Specht T, Gilbertie A, Minton W, Fang Y, Renukaradhya GJ (2015). Evaluation of humoral immune status in porcine epidemic diarrhea virus (PEDV) infected sows under field conditions. Vet Res.

[10] Diel DG, Lawson S, Okda F, Singrey A, Clement T, Fernandes MHV, Christopher-Hennings J, Nelson EA (2016). Porcine epidemic diarrhea virus: an overview of current virological and serological diagnostic methods. Virus Res.

[11] Song Y, Singh P, Nelson E, Ramamoorthy S (2016). A computationally designed serological assay for porcine epidemic diarrhea virus. J Clin Microbiol.

[12] Van Nieuwstadt AP, Zetstra T (1991). Use of two enzyme-linked immunosorbent assays to monitor antibody responses in swine with experimentally induced infection with porcine epidemic diarrhea virus. Am J Vet Res.

[13] Okda F, Liu X, Singrey A, Clement T, Nelson J, Christopher-Hennings J, Nelson EA, Lawson S (2015). Development of an indirect ELISA, blocking ELISA, fluorescent microsphere immunoassay and fluorescent focus neutralization assay for serological evaluation of exposure to north American strains of porcine epidemic diarrhea virus. BMC Vet Res.

[14] Van Diep N, Norimine J, Sueyoshi M, Lan NT, Hirai T, Yamaguchi R. US-like isolates of porcine epidemic diarrhea virus from Japanese outbreaks between 2013-2014. SpringerPlus. 2015. 10.1186/s40064-015-1552-z. 10.1186/s40064-015-1552-z PMC4668244 26693114

[15] Takahashi K, Okada K, Ohshima K (1983). An outbreak of swine diarrhea of a new-type associated with coronavirus-like particles in Japan. Jpn J Vet Sci.

[16] Sueyoshi M, Tsuda T, Yamazaki K, Yoshida K, Nakazawa M, Sato K, Minami T, Iwashita K, Watanabe M, Suzuki Y (1995). An immunohistochemical investigation of porcine epidemic diarrhoea. J Comp Pathol.

[17] Koike N, Mai TN, Shirai M, Kubo M, Hata K, Marumoto N, Watanabe S, Sasaki Y, Mitoma S, Notsu K, Okabayashi T, Wiratsudakul A, Kabali E, Norimine J, Sekiguchi S (2018). Detection of neutralizing antibody against porcine epidemic diarrhea virus in subclinically infected finishing pigs. J Vet Med Sci.

[18] Oh JS, Song DS, Yang JS, Song JY, Moon HJ, Kim TY, Park BK (2005). Comparison of an enzyme-linked immunosorbent assay with serum neutralization test for serodiagnosis of porcine epidemic diarrhea virus infection. J Vet Sci.

[19] Hou XL, Yu L, Liu J (2007). Development and evaluation of enzyme-linked immunosorbent assay based on recombinant nucleocapsid protein for detection of porcine epidemic diarrhea (PEDV) antibodies. Vet Microbiol.

[20] Gerber PF, Gong Q, Huang Y, Wang C, Holtkamp D, Opriessnig T (2014). Detection of antibodies against porcine epidemic diarrhea virus in serum and colostrum by indirect ELISA. Vet J.

[21] Gimenez-Lirola LG, Zhang J, Carrillo-Avila JA, Chen Q, Magtoto R, Poonsuk K, Baum DH, Pineyro P, Zimmerman J (2017). Reactivity of porcine epidemic diarrhea virus structural proteins to antibodies against porcine enteric coronaviruses: diagnostic implications. J Clin Microbiol.

[22] Lin CM, Gao X, Oka T, Valsova AN, Esseili MA, Wang Q, Saif LJ (2015). Antigenic relationships among porcine epidemic diarrhea virus and transmissible gastroenteritis virus strain. J Virol.

[23] Gerber PF, Lelli D, Zhang J, Strandbygaard B, Moreno A, Lavazza A, Perulli S, Botner A, Comtet L, Roche M, Pourquier P, Wang C, Opriessnig T (2016). Diagnostic evaluation of assays for detection of antibodies against porcine epidemic diarrhea virus in pigs exposed to different PEDV strains. Prev Vet Med.

[24] Simmons JH (2008). Development, application, and quality control of serology assay used for diagnostic monitoring of laboratory nonhuman primates. ILAR J.

[25] Chen Q, Thomas JT, Gimenez-Lirola LG, Hardman JM, Gao Q, Gerber PF, Opriessning T, Zheng Y, Li G, Gauger PC, Madson DM, Magstadt DR, Zhang J (2016). Evaluation of serological cross reactivity and cross neutralization between the United States porcine epidemic diarrhea virus prototype and S-INDEL-varient strains. BMC Vet Res.

[26] Schrijver RS, Kramps JA (1998). Critical factors affecting the diagnostic reliability of enzyme-linked immunosorbent assay formats. Rev Sci Tech Off Int Epiz.

[27] Mai TN, Diep NV, Yamazaki W, Okabayashi T, Mitoma S, Notsu K, Sakai Y, Yamaguchi R, Norimine J, Sekiguchi S (2018). Development of pooled testing system for porcine epidemic diarrhea using real-time fluorescent reverse-transcription loop-mediated isothermal amplification assay. BMC Vet Res.

[28] Kim C, Iseki H, Herbas MS, Yokoyama N, Suzuki H, Xuan X, Fujisaki K, Igarashi I (2007). Development of Taqman-based real-time PCR assays for diagnostic detection of *Babesia bovis* and *Babesia bigemina*. Am J Trop Med Hyg.

[29] R Studio. RStudio: integrated development environment for R (version 0.96.122) [Computer software]. Boston; 2012.

[30] Thrusfield M (2005). Diagnostic testing. Veterinary epidemiology.

