# Supplementary material for: Development of indirect enzyme-linked immunosorbent assay for detection of porcine epidemic diarrhea virus specific antibodies (IgG) in serum of naturally infected pigs
Source: BMC Vet Res. 2019 Nov 12;15:409. doi: 10.1186/s12917-019-2123-2 (PMC6852973; doi:10.1186/s12917-019-2123-2)
Supplement: Supplementary file 1 — Additional file 1: Figure S1. Evaluation for working antigen concentration of PEDV. For optimization of working PEDV antigen concentration, 3 NT positive sera (1: 128, 1:64, 1:8 of NT titer) and 3 NT negative sera (1:< 2 of NT titer) were tested at 1:100, 1:200, 1:400 and 1:800 dilutions of PEDV antigen coated ELISA plate. The serum samples were diluted at 1:1000 in this experiment. The highest OD value in NT positive sera and the lowest OD value in NT negative sera were observed for 1:100 dilution of PEDV antigen. Therefore, the working PEDV antigen concentration for the indirect ELISA was set as 1:100 dilution in this study. [file 12917_2019_2123_MOESM1_ESM.pptx]

## Slide 1
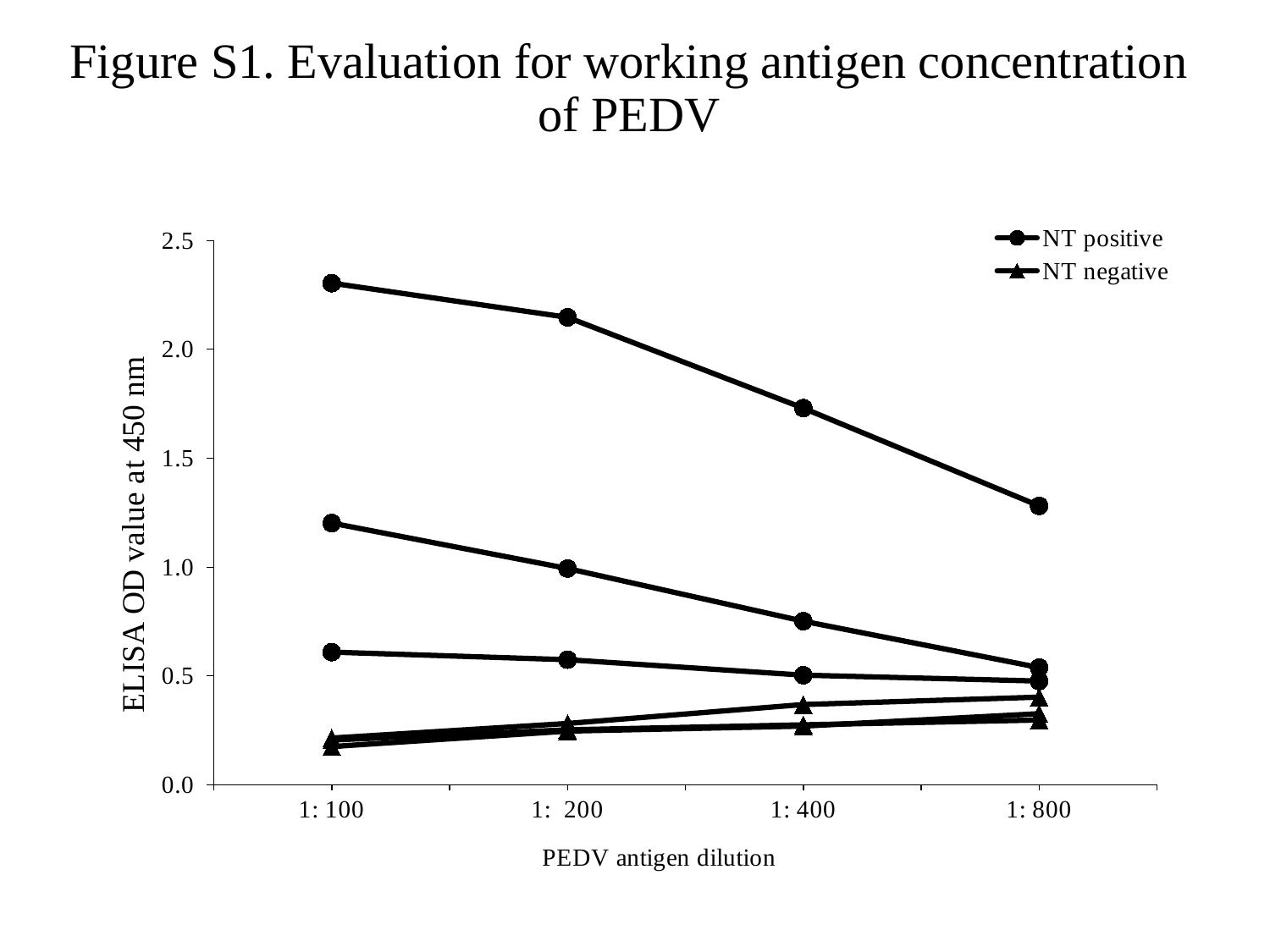

# Figure S1. Evaluation for working antigen concentration of PEDV
### Chart
| Category | NT positive | NT positive | NT positive | NT negative | NT negative | NT negative |
|---|---|---|---|---|---|---|
| 1: 100 | 2.3040000000000003 | 0.609 | 1.2020000000000002 | 0.214 | 0.205 | 0.175 |
| 1: 200 | 2.1470000000000002 | 0.574 | 0.993 | 0.281 | 0.252 | 0.24599999999999997 |
| 1: 400 | 1.73 | 0.503 | 0.751 | 0.368 | 0.275 | 0.269 |
| 1: 800 | 1.2810000000000001 | 0.47600000000000003 | 0.5379999999999999 | 0.402 | 0.29700000000000004 | 0.326 |
