# Supplementary material for: Development of indirect enzyme-linked immunosorbent assay for detection of porcine epidemic diarrhea virus specific antibodies (IgG) in serum of naturally infected pigs
Source: BMC Vet Res. 2019 Nov 12;15:409. doi: 10.1186/s12917-019-2123-2 (PMC6852973; doi:10.1186/s12917-019-2123-2)
Supplement: Supplementary file 2 — Additional file 2: Figure S2. Evaluation for working serum samples dilution. Three NT positive (1: 128, 1:64, 1:8 of NT titer) and 3 NT negative sera (1:< 2 of NT titer) were diluted to 1:62.5, 1: 125, 1: 250, 1:500, 1:1000 and, 1:2000 and tested using 1:100 diluted PEDV antigen coated plates. All NT negative sera diluted more than 1:1000 gave OD value less than 0.3, and NT positive sample with NT titer (1: 8) showed OD value higher than 0.5 in 1:1000 dilution or less. Therefore, the working serum dilution for the indirect ELISA was set as 1:1000 in this study. [file 12917_2019_2123_MOESM2_ESM.pptx]

## Slide 1
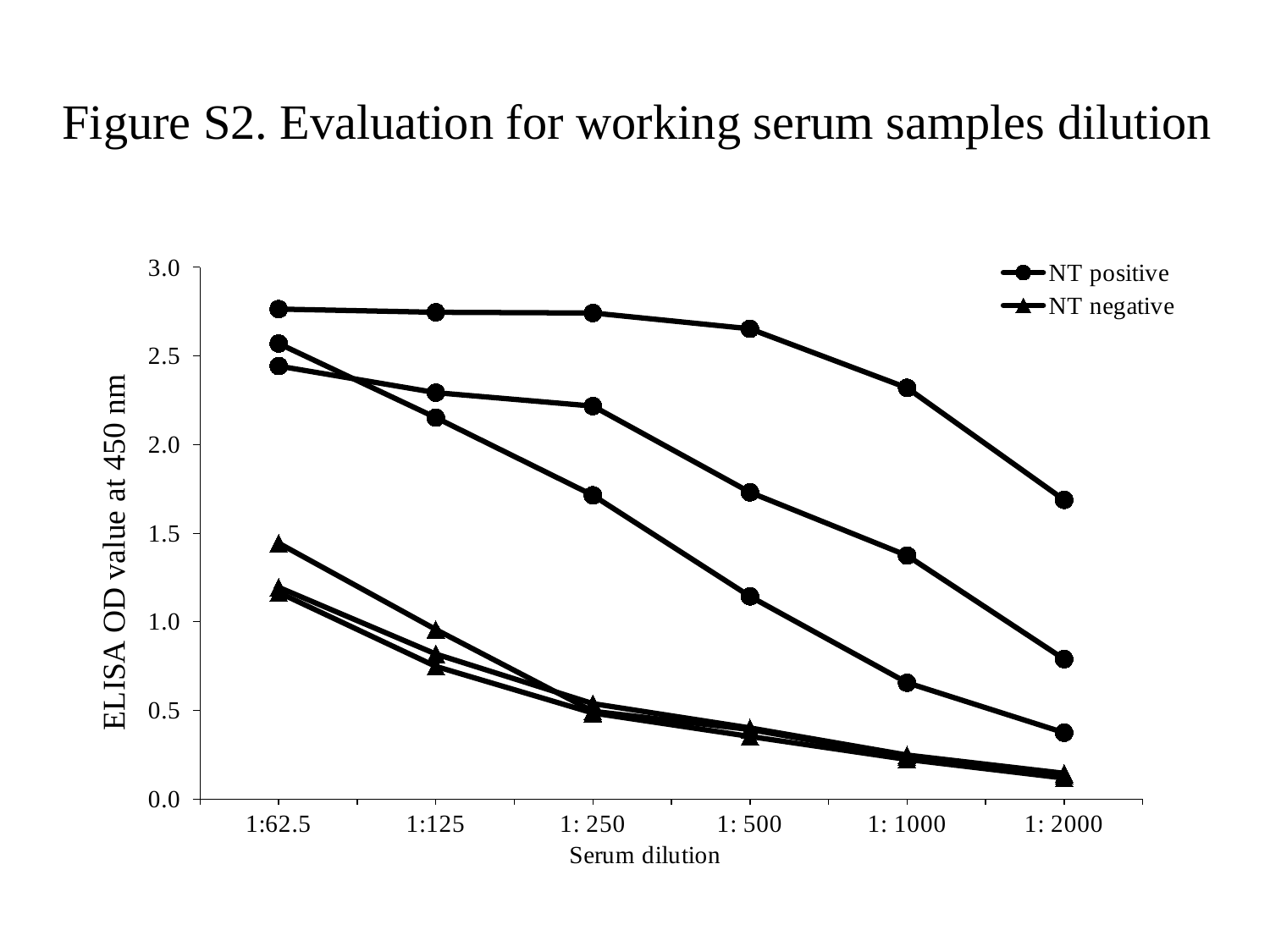

# Figure S2. Evaluation for working serum samples dilution
### Chart
| Category | NT positive | NT positive | NT positive | NT negative | NT negative | NT negative |
|---|---|---|---|---|---|---|
| 1:62.5 | 2.7640000000000002 | 2.5700000000000003 | 2.443 | 1.195 | 1.444 | 1.166 |
| 1:125 | 2.746 | 2.152 | 2.293 | 0.819 | 0.957 | 0.749 |
| 1: 250 | 2.742 | 1.714 | 2.217 | 0.5389999999999999 | 0.49600000000000005 | 0.48600000000000004 |
| 1: 500 | 2.653 | 1.1440000000000001 | 1.731 | 0.402 | 0.393 | 0.35400000000000004 |
| 1: 1000 | 2.32 | 0.657 | 1.3739999999999999 | 0.249 | 0.23800000000000002 | 0.224 |
| 1: 2000 | 1.688 | 0.375 | 0.7899999999999999 | 0.146 | 0.13299999999999998 | 0.119 |
